# GABA, but Not Bestrophin-1, Is Localized in Astroglial Processes in the Mouse Hippocampus and the Cerebellum

**DOI:** 10.3389/fnmol.2020.00135

**Published:** 2020-07-28

**Authors:** Lasse Ormel, Knut H. Lauritzen, Rainer Schreiber, Karl Kunzelmann, Vidar Gundersen

**Affiliations:** ^1^Section of Anatomy, Department of Molecular Medicine, Institute of Basic Medical Sciences, University of Oslo, Oslo, Norway; ^2^Department of Neurology, Oslo University Hospital, Ullevål, Oslo, Norway; ^3^Research Institute of Internal Medicine, Oslo University Hospital, Rikshospitalet, Oslo, Norway; ^4^Department of Physiology, University of Regensburg, Regensburg, Germany; ^5^Section for Movement Disorders, Department of Neurology, Oslo University Hospital, Rikshospitalet, Oslo, Norway

**Keywords:** astrocyte, Bergman glia, electron microscopy, rodent, immunohistochemistry

## Abstract

GABA is proposed to act as a gliotransmitter in the brain. Differences in GABA release from astroglia are thought to underlie differences in tonic inhibition between the cerebellum and the CA1 hippocampus. Here we used quantitative immunogold cytochemistry to localize and compare the levels of GABA in astroglia in these brain regions. We found that the density of GABA immunogold particles was similar in delicate processes of Bergman glia in the cerebellum and astrocytes in the CA1 hippocampus. The astrocytic GABA release is proposed to be mediated by, among others, the Ca^2+^ activated Cl^–^ channel bestrophin-1. The bestrophin-1 antibodies did not show any significant bestrophin-1 signal in the brain of wt mice, nor in bestrophin-1 knockout mice. The bestrophin-1 signal was low both on Western blots and immunofluorescence laser scanning microscopic images. These results suggest that GABA is localized in astroglia, but in similar concentrations in the cerebellum and CA1 hippocampus, and thus cannot account for differences in tonic inhibition between these brain regions. Furthermore, our data seem to suggest that the GABA release from astroglia previously observed in the hippocampus and cerebellum occurs via mechanisms other than bestrophin-1.

## Introduction

GABA is the most abundant inhibitory transmitter in the brain (Ottersen et al., 1984). It is localized in interneurons within most brain regions, and in some projecting neurons, such as in the basal ganglia. GABA is released from inhibitory nerve terminals to act on postsynaptic GABA receptors, of which there are two types; GABA_A_ receptors (GABA_A_R) and GABA_B_R receptors (GABA_B_R). GABA_A_R -mediated signaling in the brain can be divided in two components (Scimemi et al., 2005). It underlies “phasic” inhibition, which is classically fast inhibitory signaling mediated by vesicular release of GABA from presynaptic terminals onto postsynaptic GABA_A_R. “Tonic” inhibition, on the other hand, consists of continuous extrasynaptic (GABA_A_R activity that changes only slowly with the ambient neurotransmitter concentration. GABA_B_R s are metabotropic receptors, which are involved in the regulation and fine tuning of inhibitory vs. excitatory responses in the brain (Kulik et al., 2018). After release, GABA is taken up back into nerve terminals and astrocytes by the GABA transporters GAT1 and GAT3, respectively (Itouji et al., 1996; Conti et al., 2004). Like neurons, astrocytes contain GABA, both in their cell soma and their thin processes (Meinecke and Peters, 1987; Blomqvist and Broman, 1988; Gundersen et al., 2001; Le Meur et al., 2012). The prevailing view is that after astrocytes have taken up GABA, it is converted to glutamine (via catabolism to succinate and glutamate), which is then carried back into GABA terminals to replenish transmitter GABA pools (Rae et al., 2003; Holten and Gundersen, 2008; Jiang et al., 2012). However, it seems that not all astrocytic GABA is part of this GABA-glutamine cycle between neurons and astrocytes. It has been shown that GABA itself can be released from astrocytes (Christensen et al., 2018). Astrocytic GABA release can probably happen through various mechanisms (Ransom et al., 2017) such as reversal of GAT3 (Gallo et al., 1991; Barakat and Bordey, 2002) via connexin-channels (Ye et al., 2003), and through opening of volume regulated anion channels (Wang et al., 2002; Kozlov et al., 2006; Jiménez-González et al., 2011; Le Meur et al., 2012). Recently, astroglial GABA release through bestrophin-1, which is a Ca^2+^ activated chloride channel, was shown to take place in the cerebellum, accounting for tonic synaptic inhibition (Lee et al., 2010). It was shown that the brain regions prone to tonic inhibition (e.g., the cerebellum) contained much more GABA positive astroglia than the regions with little tonic inhibition (e.g., the CA1 hippocampus) (Yoon et al., 2011). Besides the cerebellum, it has been reported that the CA1 hippocampus also contains bestrophin-1 in astrocytes (Park et al., 2009).

Here, our intention was to use a well-established method for quantification of amino acids in subcellular compartments – the quantitative electron microscopic immunogold technique (Bergersen et al., 2008) to investigate the localization of GABA in delicate processes of astrocytes, and to compare the content of GABA in processes from different brain regions. Then we wanted to investigate the relation between the presence of GABA and bestrophin-1 in astroglial processes. To detect bestrophin-1 in the brain we used Western blots and confocal immunofluorescence microscopy of wild type and bestrophin-1 knock-out mice, as well as RT-PCR.

## Methods

### Antibodies

Information about the primary antibodies is given in Table 1. The primary bestrophin-1 antibodies were provided by Professor Karl Kunzelmann, University Regensburg, Germany (“Best1ab#1”) and Alan Marmorstein, Mayo Clinic, Rochester, Minnesota, United States (“Best1ab#2”). Both polyclonal rabbit anti- bestrophin-1 antibodies were raised against the peptide sequence AESYPYRDEAGTKPVLYE of the mouse protein. We used a monoclonal mouse anti-glutamine synthetase antibody against amino acids 1–373 of sheep glutamine synthetase (ab 610518, BD Biosciences), monoclonal mouse anti-GFAP clone GA5 (ab G3893, Sigma), polyclonal rabbit anti-vesicular glutamate transporter 1 (VGLUT1) antibodies raised against amino acids 456–560 of the rat protein (ab 135302, Synaptic Systems) and polyclonal rabbit anti-GABA antibodies (Gundersen et al., 2001).

**TABLE 1:** Information about the primary antibodies.

**Antibody**	**Immunogen structure**	**Species**	**Methods**	**Manufacturer**	**RRID**	**Cat. no.**
Anti GABA	GABA in complex with glutaraldehyde/formaldehyde	Rabbit, polyclonal	EM (1:300) LM (1:1000)	Raised by Jon Storm-Mathisen, (Storm-Mathisen et al., 1983)	N/A	990
Anti best1	Mouse peptide sequence AESYPYRDEAGTKPVLYE	Rabbit, polyclonal	LM (1:2000) WB (1:300–1:5000)	Raised by Karl Kunzelmann (Barro-Soria et al., 2009)	N/A	Best1ab#1
Anti best1	Mouse peptide sequence, AESYPYRDEAGTKPVLYE	Rabbit, polyclonal	LM (1:3000) WB (1:3000–1:18,000)	Raised by Alan Marmorstein	N/A	Best1ab#2
Anti GLN syntethase	amino acids 1–373 of sheep glutamine synthetase	Mouse monoclonal	EM (1:100) LM (1:800)	BD Biosciences	AB 397879	610518
Anti VGLUT1	Amino acid 456–560 of purified rat VGLUT1	Rabbit, polyclonal	WB (1:9000)	Synaptic Systems	AB 887877	135302
Anti GFAP	GFAP isolated from cow spinal cord.	Mouse, monoclonal	LM (1:500)	Sigma	AB 477010	G3893

For confocal microscopy we used secondary donkey anti-mouse and goat anti- rabbit antibodies conjugated to Alexa Fluor 488 and 568 (Invitrogen), respectively. For immunogold cytochemistry we used secondary goat anti-rabbit conjugated to 10 nm gold particles [British Biocell International (BBI)] together with goat anti-mouse conjugated to 15 nm gold particles (BBI).

The Best1ab#1 has been affinity purified, used in several immunolocalization studies and shown to be selective (Barro-Soria et al., 2008, 2009; Milenkovic et al., 2015). Its selectivity is based on the following: (1) The Best1ab#1 produced a band with an appropriate molecular weight in wild type testis, which was absent in bestrophin-1 knockout testis (see Supplementary Figure 1A in Milenkovic et al., 2015). (2) The Best1ab#1 produced a similar molecular weight band as well as staining of the retinal pigment epithelium in wild type retina, which were not present in bestrophin-1 knockout tissues (Figures 1B,D in Barro-Soria et al., 2008). (3) The Best1ab#1 produced staining of the epithelium in the proximal but not in the distal colon, and of the trachea but not the nose, corresponding to physiological differences in Ca^2+^ activated Cl^–^ currents between these regions (Barro-Soria et al., 2009). (4) Western blots of wild type mouse kidney and respiratory epithelium probed with the Best1ab#1 showed a band with an appropriate molecular weight (Barro-Soria et al., 2008, 2009) which was not present in blots of respiratory tissue from bestrophin-1 knockout mice (Barro-Soria et al., 2008). (5) The Best1ab#1 produced distinct labeling of respiratory epithelium in wild type mice, which was absent in bestrophin-1 knockout mice (Barro-Soria et al., 2008). The Best1ab#2 is a raw serum. We tested both types of bestrophin-1 antibodies on Western blots of mouse cerebellar, hippocampal, cerebral cortex and testis homogenates from adult C57bl/6 mice, as well as on Western blots of whole brain homogenates from wild type and bestrophin-1 knockout BALB/c mice. We have recently used and specificity tested the VGLUT1antibodies (Ormel et al., 2012a). The glutamine synthetase antibody has previously been used by many investigators, including us (Ormel et al., 2012a, b) and it is selective (Hammer et al., 2008).

**FIGURE 1:**
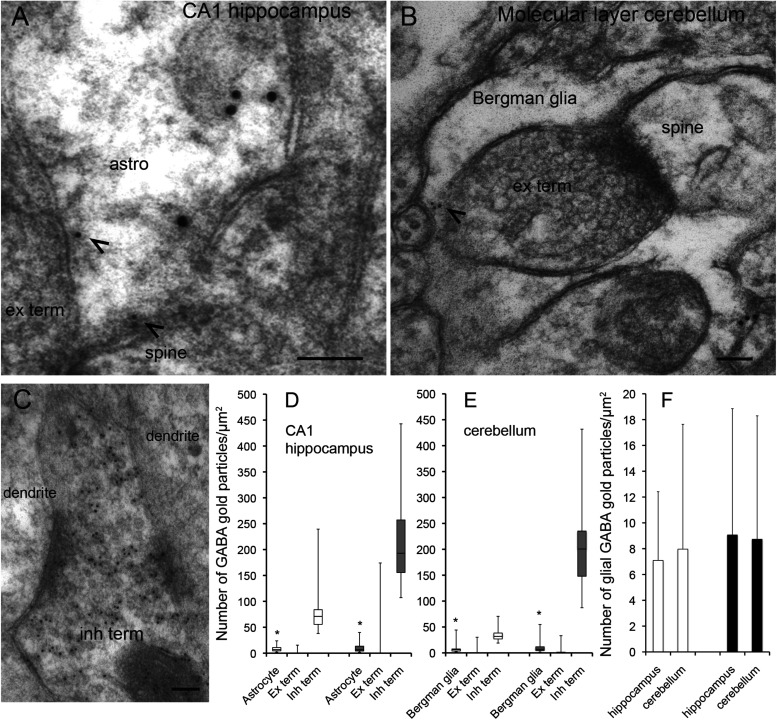
GABA is localized in CA1 hippocampal astrocytes and Bergman glia. **(A,B)** Electron micrographs showing GABA immunogold particles (small gold particles, arrowheads) in CA1 stratum radiatum astrocytes **(A)** and cerebellar Bergman glia **(B)** containing labeling for glutamine synthetase (large gold particles). The astroglial cells make contact with presynaptic terminals (ex term) forming asymmetric synapses with postsynaptic dendritic spines (spine). **(C)** GABA labeling in a nerve terminal with an inhibitory appearance (inh term) forming symmetric synapses with dendritic shafts (dendrite) in the CA1 str radiatum. Scale bars in **(A–C)**, 100 nm. **(D,E)** Quantitative representation of the density GABA immunogold particles (number of gold particles/μm^2^ in astroglial processes labeled for glutamine synthetase (Astrocyte/Bergmann glia), terminals making asymmetric synapses with dendritic spines (Ex term) and terminals making symmetric synapses with dendritic shafts (Inh term) in the CA1 hippocampus and the cerebellum). Box plots in **(C,D)** [two rats (open and filled bars)] show the lower and upper quartiles and the median. The whiskers show the minimum and the maximum density values. *The values in astrocytes and Bergman glia (astrocytes *n* = 53 and 42, Bergman glia *n* = 39 and 39) are significantly different from the values in excitatory terminals (ex term, hippocampus *n* = 90 and 96, cerebellum *n* = 85 and 67) and inhibitory terminals (inh term, hippocampus *n* = 38 and 43, cerebellum 23 and 25) (*p*< 0.05, Mann–Whitney *U*-test, two tails, SPSS). The bars in **(F)** show the mean number of GABA gold particles/μm2 ± SD in astroglia in the hippocampus and the cerebellum in the two rats presented in **(D,E)**. There was no significant difference between the GABA signal in CA1 hippocampal astrocytes and Bergmann glia (*p >* 0.05, Mann–Whitney *U*-test, two tails, SPSS).

The 990 GABA antiserum was raised in rabbits as first described in Storm-Mathisen et al. (1983). The antiserum has been specificity tested (Gundersen et al., 2001) and used in several immunolocalization studies (Bergersen et al., 2003; Gundersen et al., 2004; Morland et al., 2012). As an extra specificity precaution in the present experiments the GABA antibodies were pretreated with soluble paraformaldehyde (PFA)- and glutaraldehyde (GA) complexes of L-glutamate and β-alanine (0.2 mM of each) before applying the antibodies on the sections. Moreover, PFA-GA complexes of GABA (0.5 mM) blocked the tissue labeling of GABA.

Graded ultrathin sections contained conjugate clumps with known concentrations of GABA. The GABA conjugates were linked to brain macromolecules by glutaraldehyde/formaldehyde, freeze-dried and embedded in Durcupan ACM (Ottersen, 1987). The concentration of GABA in the embedded conjugate clumps was 0, 1.0, and 3 mM. The graded ultrathin sections were cut, mounted on nickel grids, and incubated with the GABA antibodies together with the tissue ultrathin sections (see below). The relationship between the densities of GABA immunogold particles and the concentrations of GABA in the test conjugates was used to extrapolate the approximate mM concentrations of fixed GABA in the astroglial processes in the tissue sections. Such a way of determining amino acid concentrations in tissue profiles has been performed before for GABA (Gundersen et al., 2004) glutamate (Ottersen, 1989; Bramham et al., 1990; Bergersen et al., 2012), D-aspartate (Gundersen et al., 1995), and D-serine (Bergersen et al., 2012).

### Animals

Adult male C57BL/6 mice (5–10 weeks) were anesthetized with pentobarbital (intraperitoneal, 100 mg/ml). By use of a peristaltic pump they were perfusion fixed through the heart. We used 4% PFA for bestrophin-1 immunofluorescence (*n* = 2), a mixture of 4% PFA and 0.1% GA for GABA and bestrophin-1 immunofluorescence (*n* = 1) and a mixture of 1% PFA and 2.5% GA for immunogold GABA labeling (*n* = 2)

Adult male C57BL/6 mice (5–10 weeks) were decapitated, the brains and the testis removed and placed in frozen in liquid nitrogen (*n* = 4 for Western blots). Adult male Balb/c wild type (1–5 months) and Balb/c bestrophin-1 knockout (5 months) mice were decapitated and the brains immediately dissected free. Some brains were put in 4% PFA (*n* = 2 wild type and *n* = 2 bestrophin-1 knockout brains for immunoflourescence) and immersion fixed in room temperature for 1 day and kept at 4°C until use [after 1 week the fixative was diluted 1:10 in 0.1 M sodium phosphate buffer, pH∼7.4 (PB)]. Other brains were immediately frozen in liquid nitrogen (*n* = 1 wild type brain and *n* = 1 bestrophin-1 knockout brain for Western blots, and *n* = 2 wild type brains and *n* = 1 bestrophin-1 knockout brain for RT-PCR). In all animals PFA and GA were diluted in PB. The Balb/c bestrophin-1 knockout and wild type mice were provided by A. Marmorstein. Generation of bestrophin-1 knockout mice with targeted disruption of the Vmd2 gene is described in Marmorstein et al. (2006). The mice used in this study were analyzed by Southern blot of tail DNA to identify bestrophin-1 (Vmd2) +/- heterozygotes, which then were inbred to yield Vmd2-/- homozygous mutants. The animal experiments were reviewed and approved by Norwegian Food Safety Authority (licence FOTS 3303).

### Qualitative Confocal Immunoflourescence

The fixed brains, retina and testis were cryoprotected in sucrose. 20 μm sections were cut and treated according to an immunoflourescene protocol for free floating sections (Ormel et al., 2012a, b). In brief, the sections were incubated in a blocking solution containing 2% normal calf serum (NCS) in 0.01M phosphate buffered saline (PBS) with 0.1% TritonX-100 (PBST) and subsequently incubated for 96 h with the primary antibodies. The brain sections were double labeled with rabbit anti-GABA (1:1000) and mouse anti-glutamine synthetase (1:800), or with rabbit anti-bestrophin-1 (Best1ab#1 1:100–1:2000, Best1ab#2 1:100–1:3000) and mouse anti-glutamine synthetase (1:800) or mouse anti-GFAP (1:500). The sections from the retina and the testis were single labeled with the bestrophin-1 antibodies (Best1ab#1 1:2000, Best1ab#2 1:3000). The sections were incubated in secondary goat anti- rabbit 568 (1:1000) and donkey anti-mouse 488 (1:1000) antibodies for 2 h and mounted on glass slides with Prolong Gold reagent with 4′6-diamidino-2-phenylindole (DAPI).

Z-stacks of 10 μm were obtained from the cerebellum and the stratum radiatum of CA1 hippocampus, as well as from the retina and the testis using a Zeiss LSM Pascal 5 confocal microscope at 63 times magnification (PLAN APOCHROMAT, x63, NA 1.4, oil objective). The fluorescence from the 488 and the 568 fluorophores were obtained sequentially, with a pinhole size about 1 Airy unit, and with a scan speed of 2.16 μs/pixel. They were excited sequentially with a laser wavelength of 488 and 543, and a band pass filter of 505–530 and a long pass filter of 560 obtained the emitted light, respectively. The z-stacks were obtained according to the Nyquist theorem. With an optical slice of 0.8 μm we used a slice thickness of 0.4 μm in the z-stacks. By Autoquant (MediaCybernetics)^1^ we did a blinded deconvolution of each z-stack. Long-term incubation gave a deep penetration of the sections with the primary antibodies, and thus, a rather homogeneous labeling through the sections. This made it possible to investigate fluorescent labeling deeper into the section, avoiding staining artifacts that can be present at the disrupted surface.

### Electron Microscopic Quantitative Immunogold Cytochemistry

Cerebellar and hippocampal specimens (about 1 mm^3^) fixed with 1% PFA and 2.5% GA were gradually saturated in 10, 20, and 30% glycerol to cryoprotect the tissue. Then, the tissue was embedded in Lowicryl HM20 according to a freeze embedding protocol (Bergersen et al., 2008). Ultrathin sections (gold color) of the tissue from each brain region were cut on an ultra-microtome with a diamond knife and placed on 300 mesh nickel grids and stored at room temperature. The ultrathin sections were treated with the antibodies according to an immunogold protocol (Bergersen et al., 2008). To make the epitopes more accessible to the antibodies the sections were etched in 2% H_2_O_2_ in PB. To reduce the background labeling the sections were incubated in 0.1% borohydride and 50 mM glycine in PBST. The sections were then incubated in a blocking solution with 2% human serum albumin (HSA), before the sections were incubated for 24 h at 4°C in the primary antibody solution containing rabbit no. 990 GABA antibodies (diluted 1:600) and mouse glutamine synthetase antibodies (diluted 1:100). Subsequently the sections were treated with secondary antibodies (diluted 1:20) against rabbit and mouse IgG, which were coupled to a 10 nm and a 15 nm gold particle, respectively. Each section was contrasted in uranyl acetate (2% in H_2_O) for 15 min and lead citrate (0.3% in H_2_O) for 2 min.

Random electron micrographs were taken at a primary magnification of x43000 using a Tecnai 12 FEI electron microscope with a Veleta digital camera and iTEM software (Olympus). Without viewing the gold particles 50 random electron micrographs were taken from the molecular layer of cerebellum and the stratum radiatum of CA1 hippocampus. Using a plugin for ImageJ^2^, the plasma membrane of the following profiles were outlined in all micrographs: (1) perisynaptic astroglial processes (diameter < 0.5 μm) identified by labeling for glutamine synthetase (which was used as an astrocyte marker, as it is exclusively present in astrocytes (Derouiche and Frotscher, 1991) and/or by their characteristic morphology (electron lucent appearance and concave shape), (2) terminals making symmetric synapses with dendritic shafts (inhibitory synapses), (3) terminals making asymmetric synapses with dendritic spines (excitatory synapses). The excitatory terminals were used to measure the background GABA labeling. Recorded coordinates were submitted to a program written in Python^3^ for computation of gold particle densities (number of gold particles per μm^2^) (Larsson and Broman, 2005; Larsson et al., 2011). The source code of the ImageJ plugin and the Python program is available at https://old.liu.se/medfak/forskning/larsson-max/software?l=en. Mitochondria were excluded from the quantitative analysis.

### Western Blotting

The hippocampus, the cerebellum and the testis from adult male C57BL/6 mice and the whole brain from adult male wild type and bestrophin-1 knock out Balb C mice were homogenized in 1% sodium dodecyl sulfate and Sigmafast protease inhibitor (Sigma). SDS-gel electrophoresis (10–40 μg protein per lane) was performed on polyacrylamide gels (Criterion Bio Rad 12.5% gel Tris HCl). Blots were transferred to nitrocellulose membranes, which were incubated over night with the bestrophin-1 antibodies [diluted 1:300–1:5000 (Best1ab#1), 1:2000–1:18000 (Best1ab#2)] and the VGLUT1 antibodies (diluted 1:9000). HRP conjugated secondary antibodies (diluted 1:20,000) were used to detect primary antibodies and visualized by a chemiluminescent detection system [SuperSignal West Pico Chemiluminescent Substrate (Pierce, United States)] were used to visualize immunoreactive proteins.

### Quantitative Real Time PCR

Total RNA for real-time reverse transcription polymerase chain reaction (RT-PCR) was isolated from the cerebellum of wild type and bestrophin-1 knockout Balb C mice (*n* = 2), as well as from the hippocampus of wild type Balb C mice and the testis of wild type C57BL/6 mice (*n* = 2) using RNeasy^®^ Lipid Tissue Mini Kit (Qiagen) in combination with RNase- Free DNase Set (Qiagen). RNA-quality was checked on 1% agarose gels. cDNA was produced from the isolated RNA using a High Capacity RNA-to-cDNA Kit (Applied Biosystems). The following bestrophin-1 primers were used: 5′-TCT GGG TCC AGA ACC TTC AC-3′ and 5′-TTG AAG GGA GGT CTG AGG AA-3′. These primers were designed for exon1 in the bestrophin-1 gene to correspond to the eliminated sequence in the bestrophin-1 knockout mice, which were used as negative controls.

The expression of glyceraldehyde-3-phosphate dehydrogenase (GAPDH) mRNA was used as endogenous control. The mouse GAPDH primers used were the following: 5′-TCG TCC CGT AGA CAA AAT GGT-3′- and 5′-CGC CCA ATA CGG CCA AA-3′. RT-PCR reactions were carried out in a mixture containing Power SYBR Green PCR Master Mix (Applied Biosystems), cDNA and nuclease free water. All reactions were run on a StepOne Plus Real-Time PCR system (Applied Biosystems), and analyzed using StepOne software v2.1.

All reactions were done in triplicates. Negative controls with water were performed for each target. Controls with RNA-template were used to verify that the samples did not contain genomic DNA contamination. If the Ct-value (Cycle threshold-value) was <10 between cDNA and RNA sample, the data and sample were discarded. Standard curves with a 5-point 1:10 dilution series, starting at 100 ng were performed for each target. Default PCR program settings were used. Data were calculated based on the standard curves (standard-curve method), and target of interest was normalized against a control target gene (GAPDH). Standard curves with *R*^2^ <0.99 were rejected.

## Results

The delicate perisynaptic astroglial processes (transverse diameter < 0.5 μm) are the site from which release of gliotransmitters are believed to most effectively affect neuronal activity (Gundersen et al., 2015). Here we used high resolution postembedding immunogold electron microscopy to depict GABA in these delicate astroglial processes in the hippocampus and the cerebellum. To obtain a robust immunogold signal for GABA we used tissue fixed with high glutaraldehyde (GA) and low formaldehyde (FA) (2.5% GA/1% FA). Ultrathin sections were double labeled for GABA and glutamine synthetase. In these sections we found that GABA immunogold particles were localized at high levels in nerve terminals belonging to synapses with an inhibitory appearance, but they were also present in glutamine synthetase positive delicate processes of Bergmann glia in the molecular layer of the cerebellum and of astrocytes in the CA1 stratum radiatum of the hippocampus (Figures 1A–C). Quantitative immunogold analysis revealed that the density of gold particles signaling GABA was significantly higher in astroglial processes than in nerve terminals with an excitatory appearance, but much lower than in the putative inhibitory terminals (*p*< 0.05, Mann–Whitney *U*-test, two tails, SPSS, Figures 1D,E). There was no significant difference in the density of GABA immunogold particles between delicate processes of Bergmann glia and CA1 hippocampal astrocytes (*p >* 0.05, Mann–Whitney *U*-test, two tails, SPSS, Figure 1F). Ultrathin test sections with known concentrations of GABA (see section “Methods”) incubated together with the tissue sections showed that the estimated concentration of GABA in the delicate astroglial processes in the fixed tissue was approximately 0.3 mM (see Supplementary Figure 1 for calibration curve).

As was the case for the density of GABA immunogold particles, there was no significant difference in the recorded area of the delicate astroglial processes between CA1 stratum radiatum and the cerebellar molecular layer [average total area ± SD was 32 ± 11 μm^2^ and 36 ± 14 μm^2^ (*n* = 95 processes in the hippocampus, *n* = 78 processes in the cerebellum, *p*>0.05, Mann–Witney *U*-test, two tails, SPSS)]. Also the proportion of delicate astroglial processes that contained GABA immunogold particles was similar in the hippocampus and the cerebellum. The astroglial processes with gold particle densities above those found in nerve terminals belonging to synapses with an excitatory appearance were defined as GABA positive. The proportion of GABA positive astroglial processes in the CA1 radiatum was 44% and similar to that in the cerebellar molecular layer, in which it was 46% (*n* = 95 processes in the hippocampus, *n* = 78 processes in the cerebellum, pooled data from 2 animals).

**FIGURE 2:**
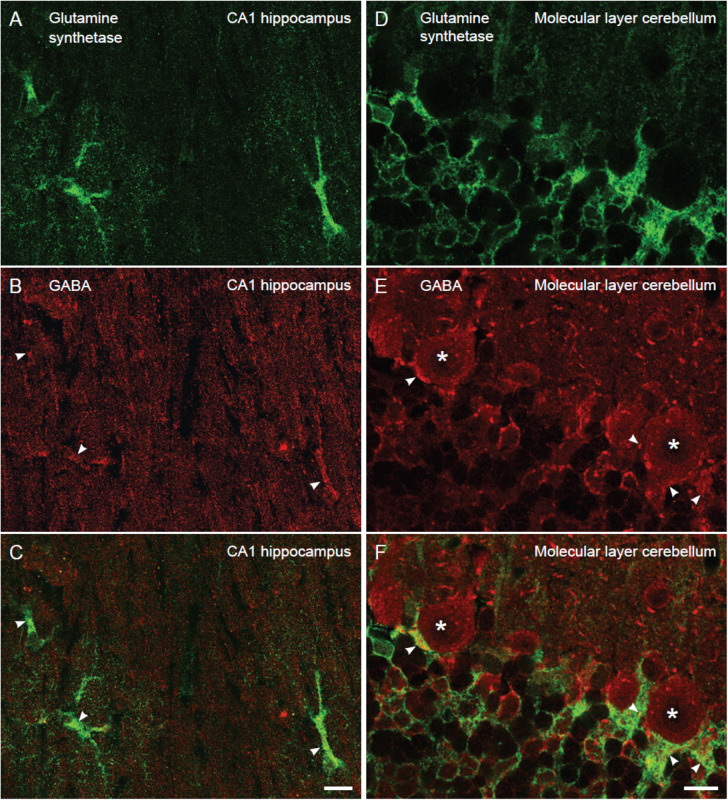
The proportion of glutamine synthetase positive astroglia containing GABA immunoreactivity is similar in the hippocampus and the cerebellum. Confocal images of sections stained for GABA (red) and astroglia (glutamine synthetase, green). In the stratum radiatum of CA1 hippocampus **(A–C)** and the Purkinje cell layer of the cerebellum **(D–F)** there are GABA positive astroglia (arrowheads, yellowish in overlay in **C,F**) at about the same number. Purkinje cells are stained for GABA (asterisk). 88% of the astrocytes contained GABA immunoreactivity in the CA1 stratum radiatum, and the value for Bergmann glia in the Purkinje cell layer of the cerebellum was 79% (*n* = 28 GABA positive astrocytes of total 32 astrocytes and 30 GABA positive Bergman glia cells of total 39 Bergmann glia). To visualize GABA and glutamine synthetase labeling in the same section we used tissue fixed by 4% FA and 0.1% GA (higher GA concentrations produce autofluorescence that disturb analysis of co-labeling), making the GABA fluorescent signal somewhat weak. We observed that the GABA staining resided within glutamine synthetase positive astroglia in z-stacks, containing slices of 0.4 μm thickness, which could be followed through astroglial cell bodies/larger processes (diameter >2 μm). Scale bars, 10 μm.

As astroglia show a strict domain localization in the brain (Bushong et al., 2002) in our electron micrographs (area of 12 μm^2^) most if not all glial processes belong to the same astrocyte. To give an approximate number of the proportion of astroglial cells in the CA1 hippocampus and the cerebellum that contained GABA we turned to confocal immunofluorescence microscopy, which allows visualization of a larger tissue area than electron microscopy. As astroglial cell bodies are localized in in the Purkinje cell layer in the cerebellum and the stratum radiatum of CA1 hippocampus, we assessed glutamine synthetase positive cell bodies and large processes (>2 μm in diameter) in these brain regions. The number of such profiles co-labeled for GABA and glutamine synthetase was recorded. We found that the percentage of GABA positive astroglia was similar in the cerebellum and the hippocampus (88 and 79% of the astroglia contained GABA immunoreactivity in the CA1 stratum radiatum and the Purkinje cell layer, respectively) (Figure 2).

**FIGURE 3:**
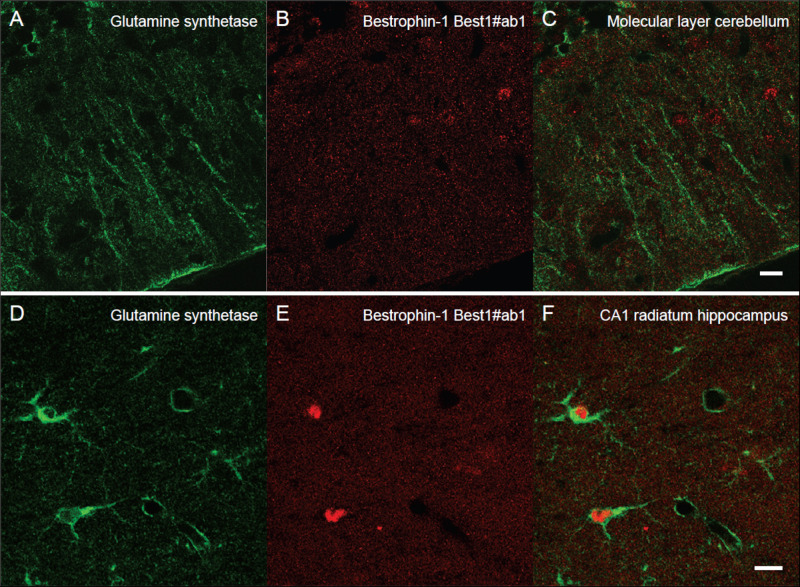
Lack of clear bestrophin-1 immunosignal in Bergman glia and CA1 hippocampal astrocytes. Confocal micrographs showing double labeling for glutamine synthetase (green, **A,D**) and for bestrophin-1 using the Best1ab#1 (red, **B,E**) in the cerebellar molecular layer **(A–C)** and the hippocampal CA1 stratum radiatum **(D–F)** of C57BL/6 mice. There is some nuclear bestrophin-1 staining (unspecific), but no clear bestrophin-1 staining of astroglial cytoplasm (overlay in **C,F**). Scale bars, 10 μm.

Next, we wanted to localize and compare bestrophin-1, a recently discovered “GABA releasing protein” (Lee et al., 2010) in astroglia between the cerebellum and the hippocampus. To this end we used double labeling immunofluorescent confocal microscopy with two types of bestrophin-1 antibodies (Best1ab#1 and Best1ab#2) and the glutamine synthetase antibodies. We first used the Best1ab#1, which has been thoroughly specificity characterized (Barro-Soria et al., 2008; Milenkovic et al., 2015). The Best1ab#1 produced a very weak and finely granulated bestrophin-1 staining pattern throughout in the neuropil (Figure 3), very much like the labeling pattern we observed when we omitted the primary antibodies (not shown). This was the case both in the cerebellum and in the CA1 hippocampus (Figure 3). We could not find any convincing sign of overlapping fluorescence signals of bestrophin-1 and glutamine synthetase (Figure 3). We have observed more than a total of 1000 astroglial profiles of the hippocampus and the cerebellum stained for bestrophin-1 in the confocal microscope, but not seen any clear signs of bestrophin-1 labeling in astroglia. Likewise, the Best1ab#2 produced weak neuropil labeling and there was not any significant astroglial labeling, neither in the cerebellum nor in the CA1 hippocampus (Supplementary Figure 2). Then we checked the bestrophin-1 staining using bestrophin-1 knock out mice as negative controls. When comparing bestrophin-1 labeling in the cerebellum and the CA1 hippocampus between wild type and bestrophin-1 knockout brains, the labeling was weak and we could not detect any significant difference in the bestrophin-1 labeling patterns (Supplementary Figures 3, 4). Bestrophin-1 has been detected at high levels in the testis and the retina (Krämer et al., 2004; Marmorstein et al., 2006; Barro-Soria et al., 2008; Milenkovic et al., 2015). Therefore, we used these tissues as positive controls of the bestrophin-1 staining in the brain. In the testis and the retina both types of bestrophin-1 antibodies did produce strong and distinct labeling of interstitial cells and retinal pigment epithelium, respectively (Supplementary Figure 5; for lower power images of the bestrophin-1 staining in the retina and the testis, see Supplementary Figures 6, 7). In addition, in all types of tissue examined, the Best1ab#1 gave staining of nuclei belonging to different types of cell and the Best1ab#2 antibodies gave a somewhat similar pattern, as well as a course staining pattern in patches, which we regard as non-specific staining (as it was present also in bestrophin-1 knockout tissues).

**FIGURE 4:**
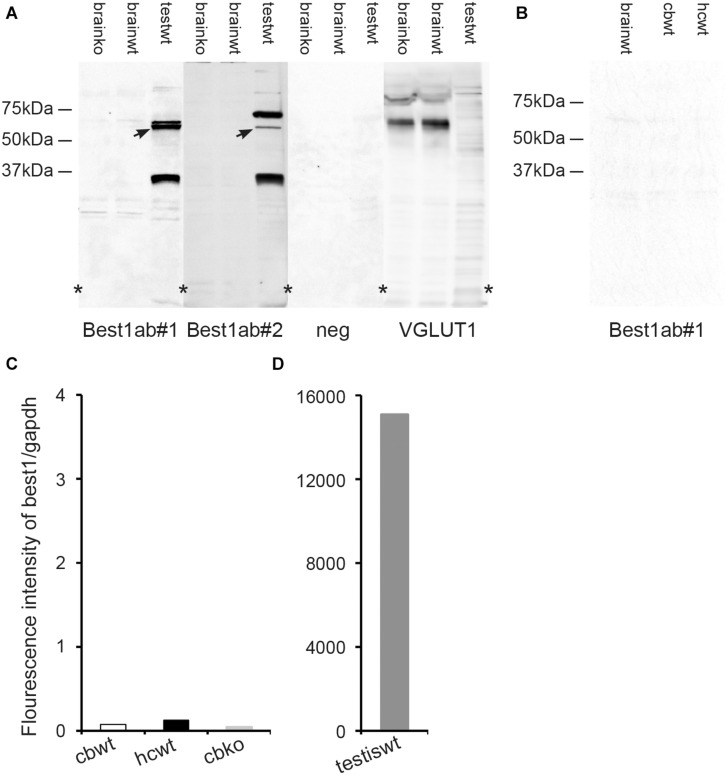
The brain expression of bestrophin-1 is very low. **(A)** Western blots of the whole brain (20 μg protein per lane) from wild type (brainwt) and bestrophin-1 knockout (brainko) Balb/c mice, and from the wild type testis labeled with two types of antibodies against bestrophin-1 [Best1ab#1 (1:1000), Best1ab#2 (1:9000), and with antibodies against VGLUT1 (1:9000)]. Note that the bestrophin-1 antibodies do not produce any bands in the brain, while in the testis (testwt) they give a band at about 64 kDa (arrows) (corresponding to the molecular weight of mouse bestrophin-1; Bakall et al., 2003). **(B)** Western blots of the whole brain (brainwt), the cerebellum (cbwt) and the hippocampus (hipwt) (20 μg protein per lane) from C57BL/6 mice labeled with antibodies against bestrophin-1 [Best1ab#1(1:1000)]. **(C,D)** Expression profile of bestrophin-1 mRNA. Quantitative RT-PCR of wild type tissue from the cerebellum (cbwt) and the hippocampus (hipwt), as well as of the bestrophin-1 knockout cerebellum (cbko), and the wild type testis (testiswt). Asterisks: denote splicing marks and that the blots are taken from entire original gels (see Supplementary Figures 8–11).

As an additional control of the Best1ab#1 and Best1ab#2 labeling described above we made Western blots of whole brain homogenates from wild type and bestrophin-1 knockout mice (Balb/c). Blots probed with the Best1ab#1 and Best1ab#2 produced very weak bands, which were similar in the wild type and the bestrophin-1 knockout brain (Figure 4A). Likewise, Western blots of the hippocampus and the cerebellum from C57Bl/6 mice did not show any evidence of specific bestrophin-1 bands (Figure 4B). However, in line with the immunofluorescence results Western blots of the testis labeled with the Best1ab#1 and the Best1ab#2 showed a band with the appropriate molecular weight of about 64 kDa (Figure 4A) suggesting that both types of bestrophin-1 antibodies recognise the bestrophin-1 protein. As a control of our Western blot method and the integrity of the tissue homogenates, we labeled the whole brain Western blots described above with antibodies against VGLUT1. As anticipated, these antibodies produced a strong VGLUT1 band, both in the wild type and the knockout tissue, but no specific bands in the testis (Figure 4A).

To detect bestrophin-1 in the brain with a method that is independent of antibody labeling we used quantitative RT-PCR. In the hippocampus and the cerebellum the bestrophin-1 mRNA expression levels were very low. In the cerebellum the bestrophin-1 mRNA expression in the wild type mice was similar to the expression in bestrophin-1 knockout mice (Figure 4C), indicating that the bestrophin-1 mRNA level in the wild type brain represent a background signal. Bestrophin-1 was, however, highly expressed in the testis (Figure 4D), demonstrating well-functioning RT-PCR primers and that the Western and the immunofluorescence data are valid.

## Discussion

Here, we give evidence that GABA is localized in cerebellar Bergmann glia and in CA1 hippocampal astrocytes. The GABA content in these delicate astroglial processes, as revealed by immunogold quantifications, is low, but similar in the molecular layer of the cerebellum and the stratum radiatum of the CA1 hippocampus. Thus, we could not find any firm evidence of differences in the distribution of GABA in astroglia between these two brain regions. Likewise, for other astroglial parameters, such as the recorded area of astroglial processes and the proportion of GABA positive astroglia, there were no difference between the hippocampus and the cerebellum.

These findings are in contrast to previous results reporting strong GABA labeling of Bergmann glia in the cerebellum, but almost absent in astrocytes the CA1 hippocampus (Lee et al., 2010; Yoon et al., 2011). The main difference between our GABA results and the latter is that we used tissue fixed for optimal immunocytochemical GABA visualization (high glutaraldehyde/low formaldehyde), while they used a fixative containing 4% formaldehyde and no glutaraldehyde. It has been shown that in brain tissue fixed with 2.5% glutaraldehyde/1% formaldehyde the amount of free amino acids retained in the tissue is about 50%, while in tissue fixed only with 4% formaldehyde only about 5% of the free amino acid is retained. This means that the GABA immunosignal in tissue fixed with high glutaraldehyde (as was the case in the present study) is much stronger than that in tissue fixed with no glutaraldehyde and high formaldehyde (as was the case in Lee et al., 2010; Yoon et al., 2011). Indeed, in our lab when we label brain tissue fixed with 4% FA only, the GABA immunosignal is very weak (LO and VG, unpublished result). In this respect, it should be mentioned that preembedding immune-electron microscopic results of Le Meur et al. (2012) are in line with ours, as they found similar amounts of GABA positive astrocytes in the “strong tonic inhibitory” CA1/CA3 hippocampus and the “weak tonic inhibitory” dentate gyrus. The latter investigators used high glutaraldehyde (2%) to fix the tissue. To be noted is that our GABA immunogold result in astrocytes in the CA1 hippocampus is in good agreement with what our group has previously reported (Gundersen et al., 2001). The density of GABA immunogold particles in astrocyte processes was about 20-fold less than in putative inhibitory nerve terminals both in this study and in Gundersen et al. (2001).

By using a well characterized system for immunogold semi-quantification of amino acid concentrations in brain tissue (Ottersen, 1987, 1989) we found that the concentration of GABA in astroglia was well below 1 mM. This finding is in contrast to the results of the immunogold study by Yoon et al. (2014) who estimated the level of GABA in Bergman glia cells to be 5–10 mM. This estimation is not precise, as it was based upon a relative difference of 1/10 in gold particle density of GABA between Bergman glia cells and nearby GABAergic terminals in the molecular layer, and an assumption that the level of GABA in the molecular layer GABAergic terminals was the same as in inhibitory Purkinje axon terminals. The latter terminals have by indirect detection methods been estimated to contain approximately 50–100 mM (Fonnum and Walberg, 1973). In Yoon et al. (2014) the brain tissue was fixed in 4% paraformaldehyde and 0.01 or 0.5% glutaraldehyde. Again, this may be too low glutaraldehyde for optimal GABA labeling, since it will only retain about 20% of tissue free amino acids (Storm-Mathisen and Ottersen, 1990). It should also be mentioned that previous light microscopic immunoperoxidase staining, using GABA antibodies, which were thoroughly specificity tested, have shown predominantly neuronal staining and no clear sign of labeling of the characteristic radial Bergmann glia in the Purkinje and molecular layer in the cerebellum (Ottersen et al., 1988; Figures 1B,D). This is similar to the immunofluorescent GABA staining pattern presented here (Figure 2). This raises the question of how reliable is the GABA labeling pattern presented by Lee et al., 2010; Yoon et al., 2011, 2014 and whether the rather strong GABA radial labeling in the cerebellar Bergmann glia in these studies represents unspecific staining.

When it comes to the bestrophin-1 data we think that these support the notion that bestrophin-1 is present in the brain at concentrations below the limits of our protein and mRNA detection methods. This is based on the following: (1) Using wild type and bestrophin-1 knockout brains the bestrophin-1 antibodies failed to show any specific bestrophin-1 protein signal in the brain, but produced a distinct signal in the retinal pigment epithelium and the testis. (2) The bestrophin-1 mRNA expression levels in the brain were very low and similar in wild type and bestrophin-1 knock out brain. Therefore, we believe that the present confocal immunofluorescent results allow us to draw the conclusion that bestrophin-1 is present at very low concentrations in astroglia in the cerebellum and the hippocampus. The reason for the contradiction between our results and those reported by Park et al. (2009), Lee et al. (2010), Yoon et al. (2011), Woo et al. (2012) is unclear. In the mouse there are not many reports on microscopic expression of bestrophin-1 in the brain except from the above mentioned publications. However, one microscopic study in the developing mouse brain showed evidence of bestrophin-1 in the meninges and the choroid plexus, but not in the brain tissue in the cortical plate (Tochitani and Kondo, 2013) which seems to be in agreement with our results. Supporting our notion of using the retina and the testis as positive control tissues, the bestrophin-1 protein has been found to be microscopically located in the mouse retinal pigment epithelium (Bakall et al., 2003; Marmorstein et al., 2006; Milenkovic et al., 2015) while bestrophin-1 mRNA is very strongly expressed in the testis (Krämer et al., 2004). Additional evidence for the presence of bestrophin-1 in the testis and the retina has been given in a bestrophin-1-cre transgenic mice, which showed a recombination signal the retinal pigment epithelium as well as in the testis (Iacovelli et al., 2011). Furthermore, using *in situ* hybridization in mouse tissue, Petrukhin et al. (1998) reported that they could only detect labeling of the retinal pigment epithelium and the testis, and not in any other tissue. In human, the latter study showed strong mRNA expression by Northern blotting in the retinal pigment epithelium, and a weak signal in the brain (Petrukhin et al., 1998). Another human study, using the same method, showed bestrophin-1 expression exclusively in the retinal pigment epithelium and no signal in the brain (Marquardt et al., 1998). One study in canine reported strong mRNA expression by RT-PCR in the retinal pigment epithelium/choroid plexus, but only low expression in brain and the retina (Guziewicz et al., 2007). Interestingly, in line with the present findings, Western blots of porcine tissues showed no bestrophin-1 band in brain tissue, but strong labeling of the retinal pigment epithelium (Stanton et al., 2006; Marmorstein et al., 2009). Thus, across different species many previous studies could not detect any strong expression of bestrophin-1 in brain tissue. This is in line with our bestrophin-1 results. The Best1ab#1 produced a band signaling bestrophin-1 on Western blots of testis in wild type mice (Milenkovic et al., 2015), but not in bestrophin-1 knockout mice (Milenkovic et al., 2015), as well as giving distinct microscopic staining of interstitial cells in the testis. In the retina the Best1ab#1 produced staining of the retinal pigment epithelium in wild type mice (Barro-Soria et al., 2008), which was absent in bestrophin-1 knockout mice (Barro-Soria et al., 2008). Thus, we believe that we can conclude that the bestrophin-1 antibodies recognize the bestrophin-1 protein. This suggests that the negative staining with the bestrophin-1 antibodies in the brain is caused by a low bestrophin-1 concentration and not by the possibility that the bestrophin-1 antibodies do not recognize the bestrophin-1 protein.

Concerning the ability of bestrophin-1 to flux GABA some critical points may be made. Bestrophin-1 is a Cl^–^ channel that is permeable for anions (Sun et al., 2002). In addition, bestrophin-1 has shown to flux both GABA, aspartate and glutamate with low permeabilities compared to Cl^–^ (aspartate about 0.2 (Qu et al., 2003), glutamate about 0.5 (Park et al., 2009), GABA about 0.3 (Lee et al., 2010). Opposing these results are data from chicken bestrophin-1, which has been shown to be impermeable to glutamate and aspartate (Kane Dickson et al., 2014; Vaisey et al., 2016). Based on the structural properties of the bestrophin pore, which was found to be very narrow, it was questioned if it will allow permeation of large amino acids (Kane Dickson et al., 2014; Yang et al., 2014; Vaisey et al., 2016; for discussion see Kunzelmann, 2015). In contrast to these X-ray based studies, a recent cryo-electron microscopy study of chicken bestrophin-1 indicated that the open channel conformation has a widened neck, making it possible that slow conductance of larger solutes can take place (Miller et al., 2019). In the present study we estimated the concentration of GABA to be below 1 mM in both hippocampal and Bergmann astroglial processes. The concentration of glutamate and aspartate in the astrocytic cytosol in the CA1 hippocampus is somewhat higher and about 2 mM (Gundersen et al., 1998, 2001). Previous immunogold studies of glutamate in the cerebellum (Somogyi et al., 1986) and the hippocampus (Bramham et al., 1990) have shown that, like for GABA, the concentration of glutamate is similar in Bergmann glia and CA1 hippocampal astrocytes (the glutamate concentration in astroglia is about 20% of that in glutamatergic nerve terminals). Thus, opening of bestrophin-1 should therefore lead to competitive efflux of all of these amino acids. Therefore, the selective bestrophin-1 mediated release of glutamate reported in the CA1 hippocampus (Yoon et al., 2011; Han et al., 2013) is not easily reconciled with our findings on the GABA content in the CA1 hippocampus and cerebellar astroglia.

The source of GABA mediating tonic inhibition is not clear. In the cerebellum it may come from spillover of GABA released from other sources than bestrophin-1, e.g., nearby synapses as proposed by Diaz et al. (2012) who could not find evidence that bestrophin-1 contributes to tonic GABAergic currents in the cerebellum. Moreover, it has been shown that granule cell GABA_A_ receptors are activated both tonically and by GABA spillover, including acetylcholine-mediated inhibition (Hamann et al., 2002). In the hippocampus, Glykys and Mody (2007) showed that tonic and phasic inhibitory currents were highly correlated under both increased and reduced vesicular GABA release, making the authors conclude that most of tonic inhibition in the hippocampus is derived from vesicular GABA release.

Taken together, our results suggest that GABA is indeed present in astroglia in the CA1 hippocampus and the cerebellum, but in equal levels, meaning that different GABA levels in hippocampal and cerebellar astroglia cannot easily account for differences in tonic inhibition between these regions. Moreover, our results indicate that bestrohin-1 is probably not responsible for the main part of tonic inhibitory currents in the cerebellum and the hippocampus.

## Data Availability Statement

All datasets generated for this study are included in the article/Supplementary Material.

## Ethics Statement

The animal study was reviewed and approved by the Norwegian Food Safety Authority PO Box 383 2381 Brumunddal Norway.

## Author Contributions

LO: planning and performing the immunocytochemical and Western blot experiments, analyzing the data, and writing the manuscript. KL: performing the PCR experiments, analyzing the data, and writing the manuscript. RS and KK: analyzing the data and writing the manuscript. VG: supervising LO, designing and planning the experiments, analyzing the data, and writing the manuscript. All authors contributed to the article and approved the submitted version.

## Conflict of Interest

The authors declare that the research was conducted in the absence of any commercial or financial relationships that could be construed as a potential conflict of interest.

## Footnotes

1 www.mediacy.com

2 http://rsb.info.nih.gov/ij/

3 http://www.python.org
