# Supplementary material for: GABA, but Not Bestrophin-1, Is Localized in Astroglial Processes in the Mouse Hippocampus and the Cerebellum
Source: Front Mol Neurosci. 2020 Jul 28;13:135. doi: 10.3389/fnmol.2020.00135 (PMC7399226; doi:10.3389/fnmol.2020.00135)
Supplement: Supplementary file 1 [file Data_Sheet_1.docx]

Supplementary Figure 1

Relationship between GABA gold particle density and concentration of fixed GABA as assessed by means of a model section with known concentrations of GABA. The slope and y-intercept was determined by regression analysis (Exel) and the R2 value is indicated. A linear regression was assumed. Each data point gives the mean number of gold particles/µm2 ±SD calculated over conjugates from 4-5 electron micrographs.

Supplementary Figure 2

Bestrophin-1 immunosignal in Bergman glia and CA1 hippocmpal astrocytes using the Best1ab#2. Confocal micrographs showing double staining for astroglia (glutamine synthetase, green) and bestrophin-1 (red) in the cerebellar molecular layer (A-C) and the CA1 stratum radiatum (D-F) of C57BL/6 mice. There is no significant bestrophin-1 staining of astroglial cells (overlay in C and F), but some staining of nuclear and neuronal dendritic profiles. Scale bars 10 μm.

Supplementary Figure 3

Using the Best1ab#1 we could not detect any difference in the bestrophin-1 labelling between wild type and bestrophin-1 knockout brains. Confocal images show bestrophin-1 labelling (red) and astrocyte labelling (GFAP, green). There is some very weak and scattered labelling for bestrophin-1, some of which could be ascribed to astroglia (arrowheads), both in the wild type cerebellum (wt cb, A-C) and the hippocampus (wt hc, G-I), as well as in the bestrophin-1 knockout cerebellum (best1 KO cb, D-F) and hippocampus (best1 KO hc, J-L). Images are from Balb/c mice. Scale bars, 10 μm.

Supplementary Figure 4

Using the Best1ab#2 we could not detect any difference in the bestrophin-1 labelling between wild type and bestrophin-1 knockout brains. Confocal images show bestrophin-1 labelling (red) and astrocyte labelling (GFAP, green). There was no clear sign of bestrophin-1 staining in astroglia, neither in the cerebellum (A-C (wild type, wt cb), D-F (bestrophin-1 knock out, best1 KO cb)), nor in the hippocampus (G-I (wild type, wt hc), J-L (bestrophin-1 knockout, best1 KO hc)). Images are from Balb/c mice. Scale bars, 10 μm.

Supplementary Figure 5

The bestrophin-1 antibodies recognise bestrophin-1 in the retina and the testis. Both the Best1ab#1(A and C) and the Best1ab#2 (B and D) stain the retinal pigment epithelium (RPE, arrowheads) (A and B) and interstitial cells (arrows) in the testis, leaving the seminiferous tubules (asterisk) unstained (C and D). Images are from C57BL/6 mice. For lower power images of the bestrophin-1 staining in the retina and the testis, see Supplementary Figure 6, 7. Scale bars 10 µm.
