# Supplementary figures and images for: GABA, but Not Bestrophin-1, Is Localized in Astroglial Processes in the Mouse Hippocampus and the Cerebellum
Source: Front Mol Neurosci. 2020 Jul 28;13:135. doi: 10.3389/fnmol.2020.00135 (PMC7399226; doi:10.3389/fnmol.2020.00135)

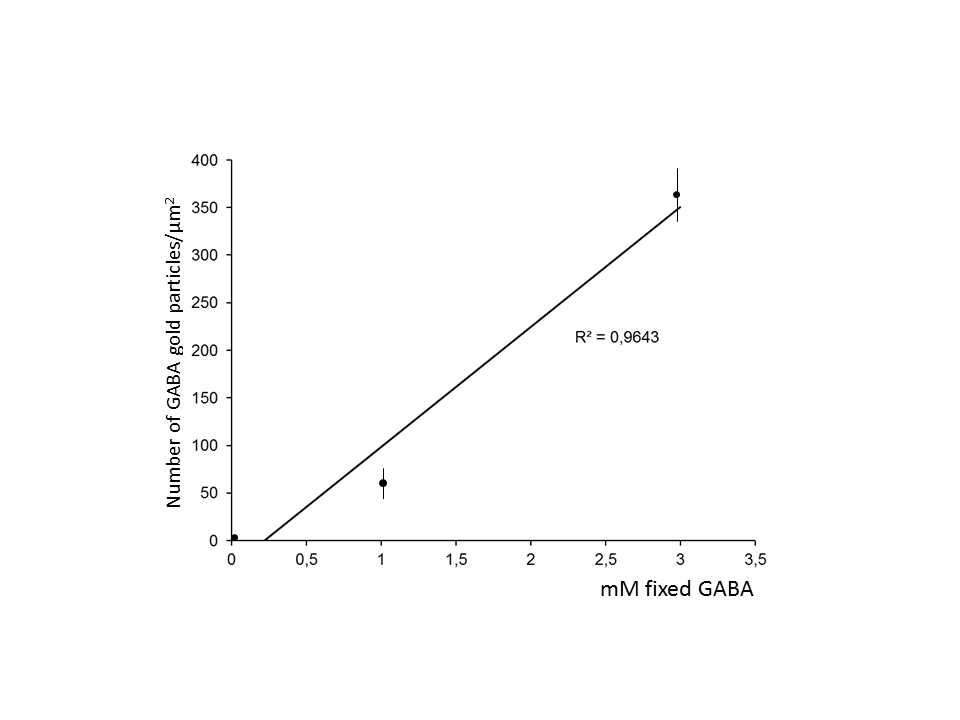

Supplement: Supplementary file 2 [file Image_1.TIF]

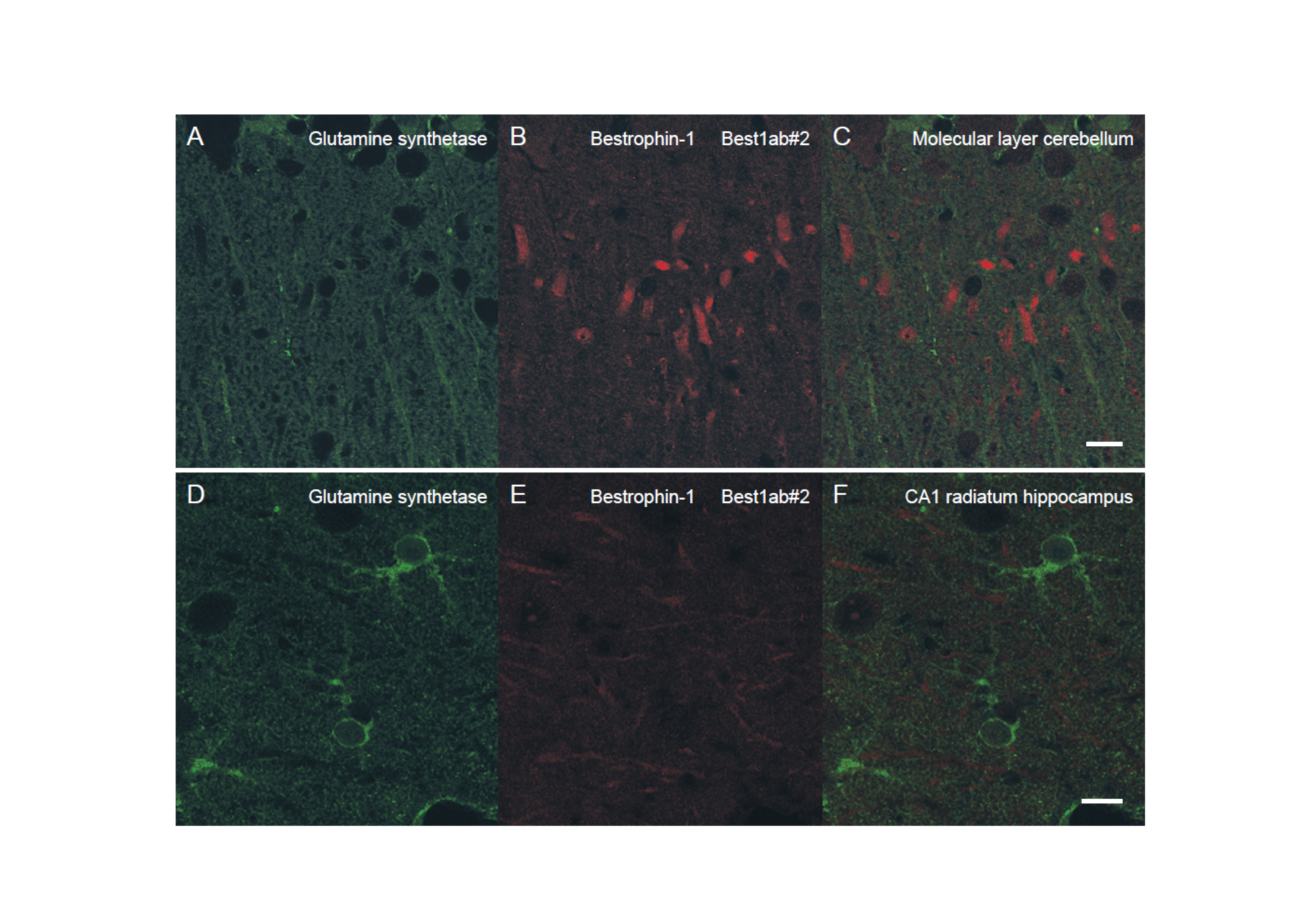

Supplement: Supplementary file 3 [file Image_2.TIF]

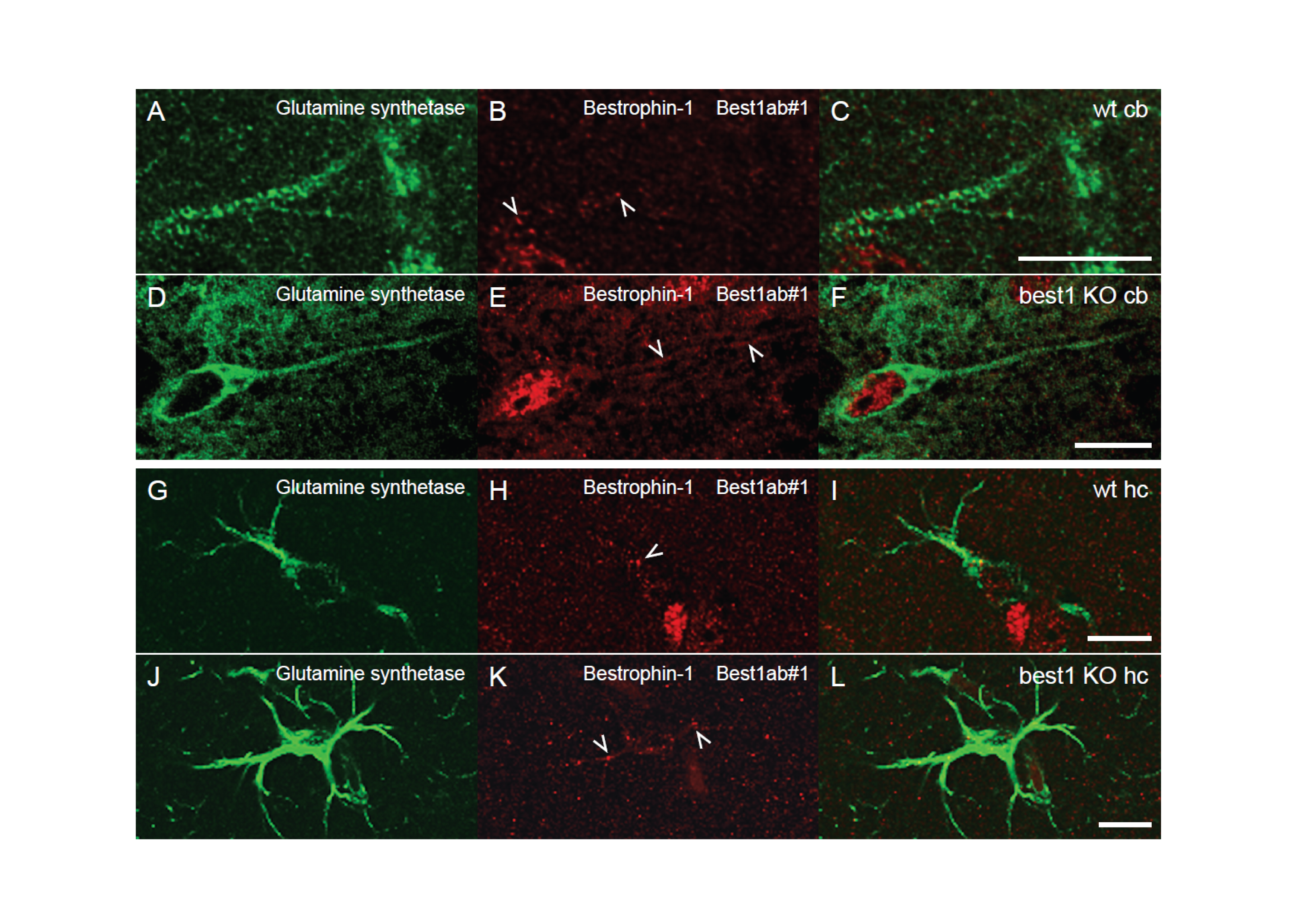

Supplement: Supplementary file 4 [file Image_3.TIF]

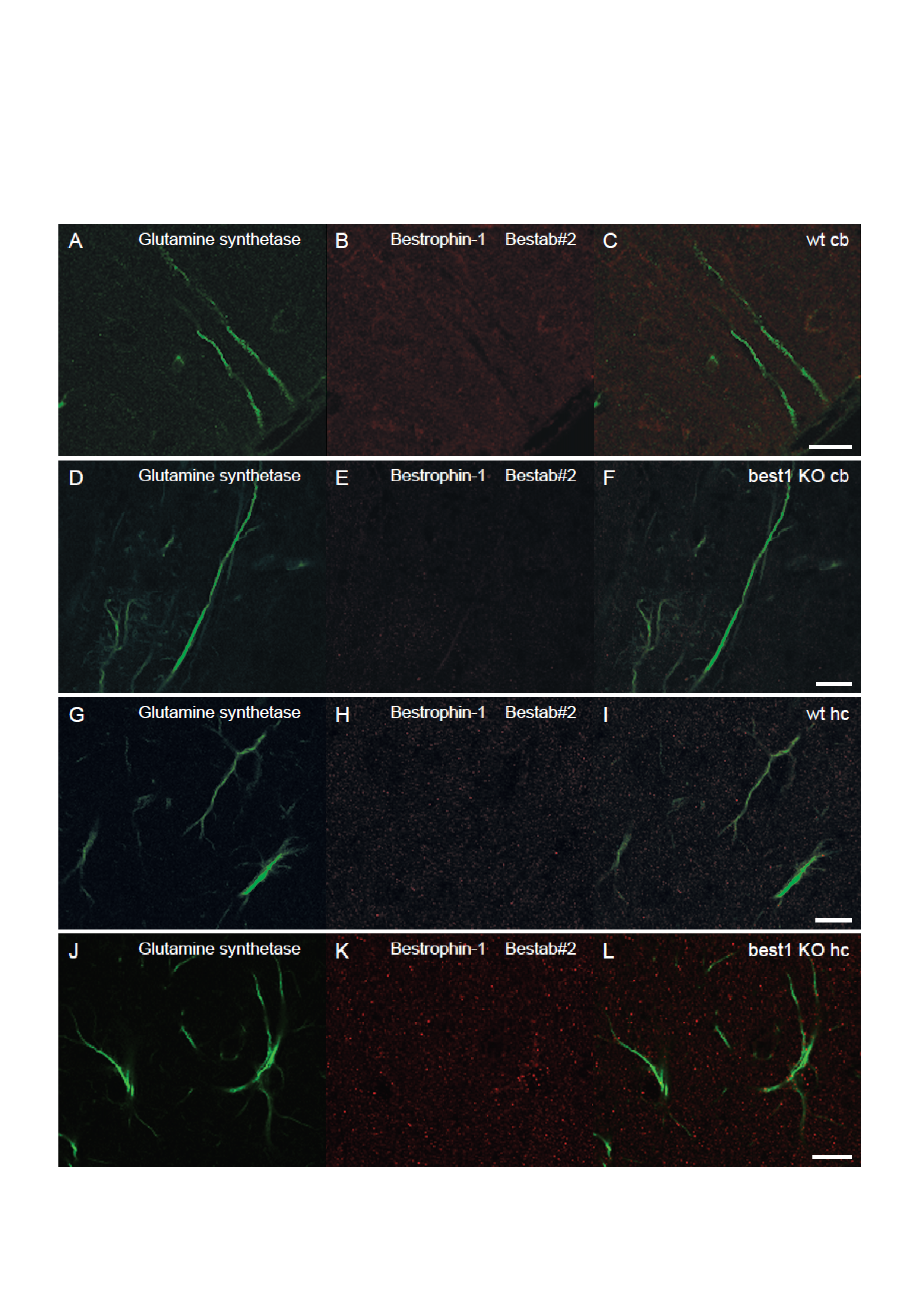

Supplement: Supplementary file 5 [file Image_4.TIF]

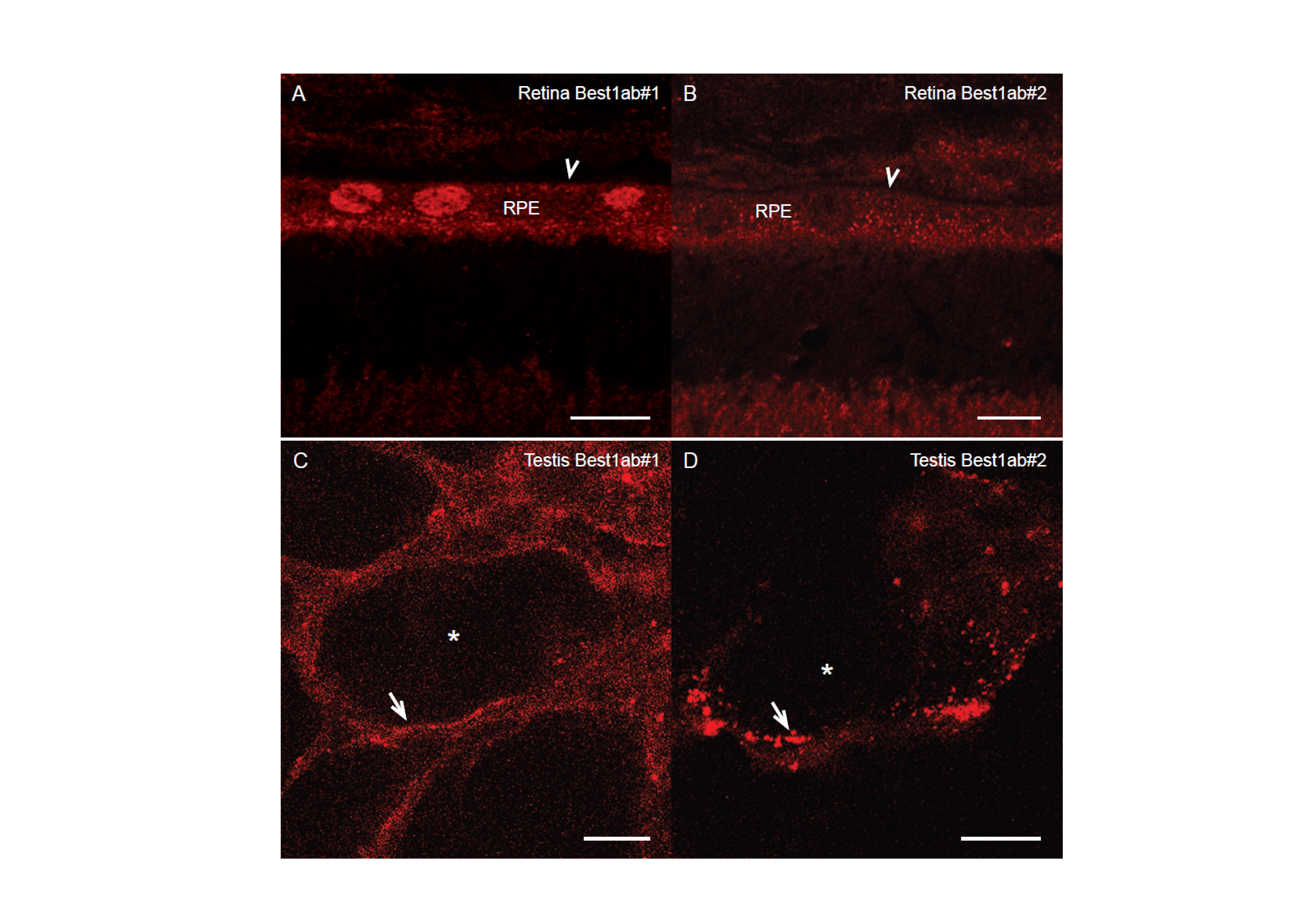

Supplement: Supplementary file 6 [file Image_5.TIF]

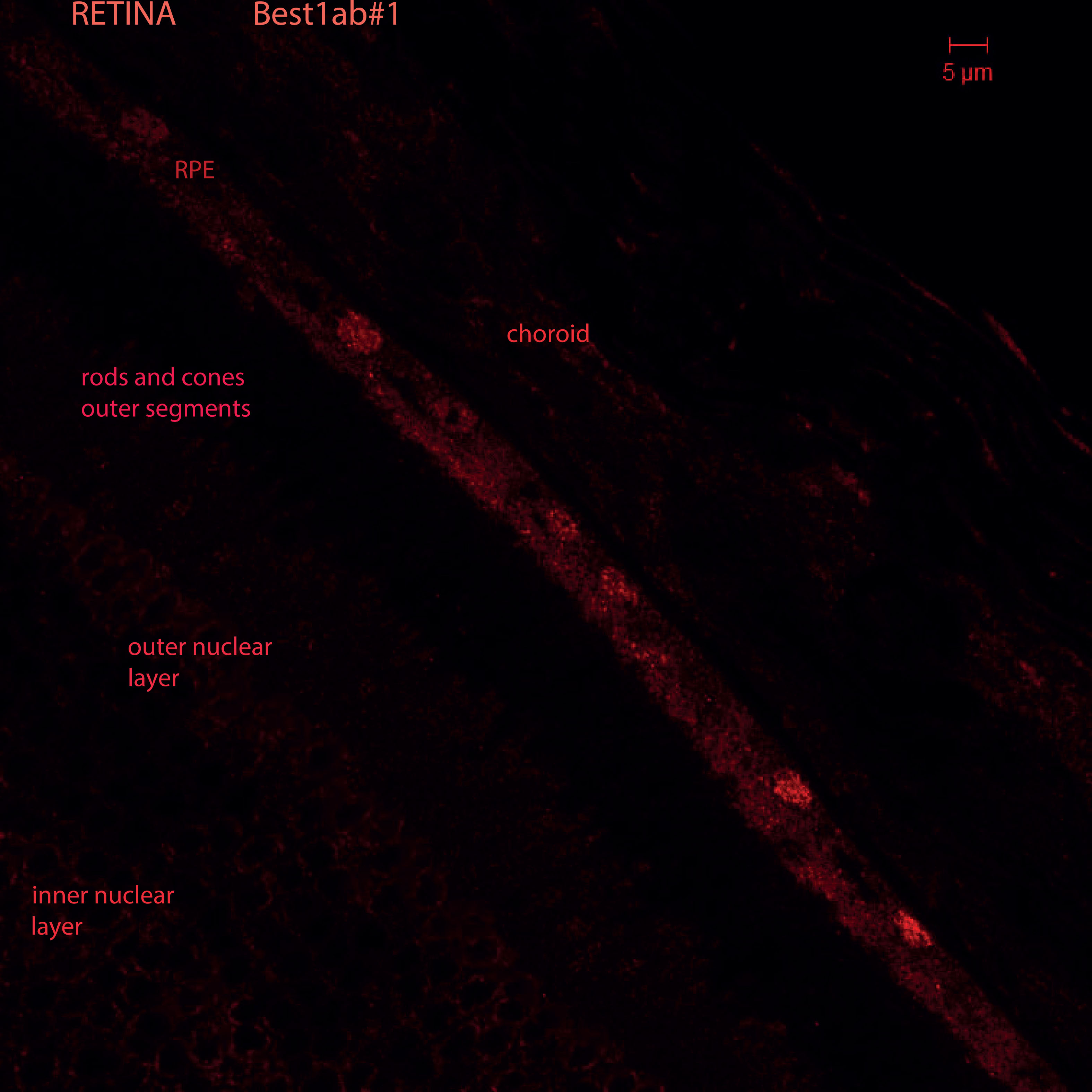

Supplement: Supplementary file 7 [file Image_6.jpeg]

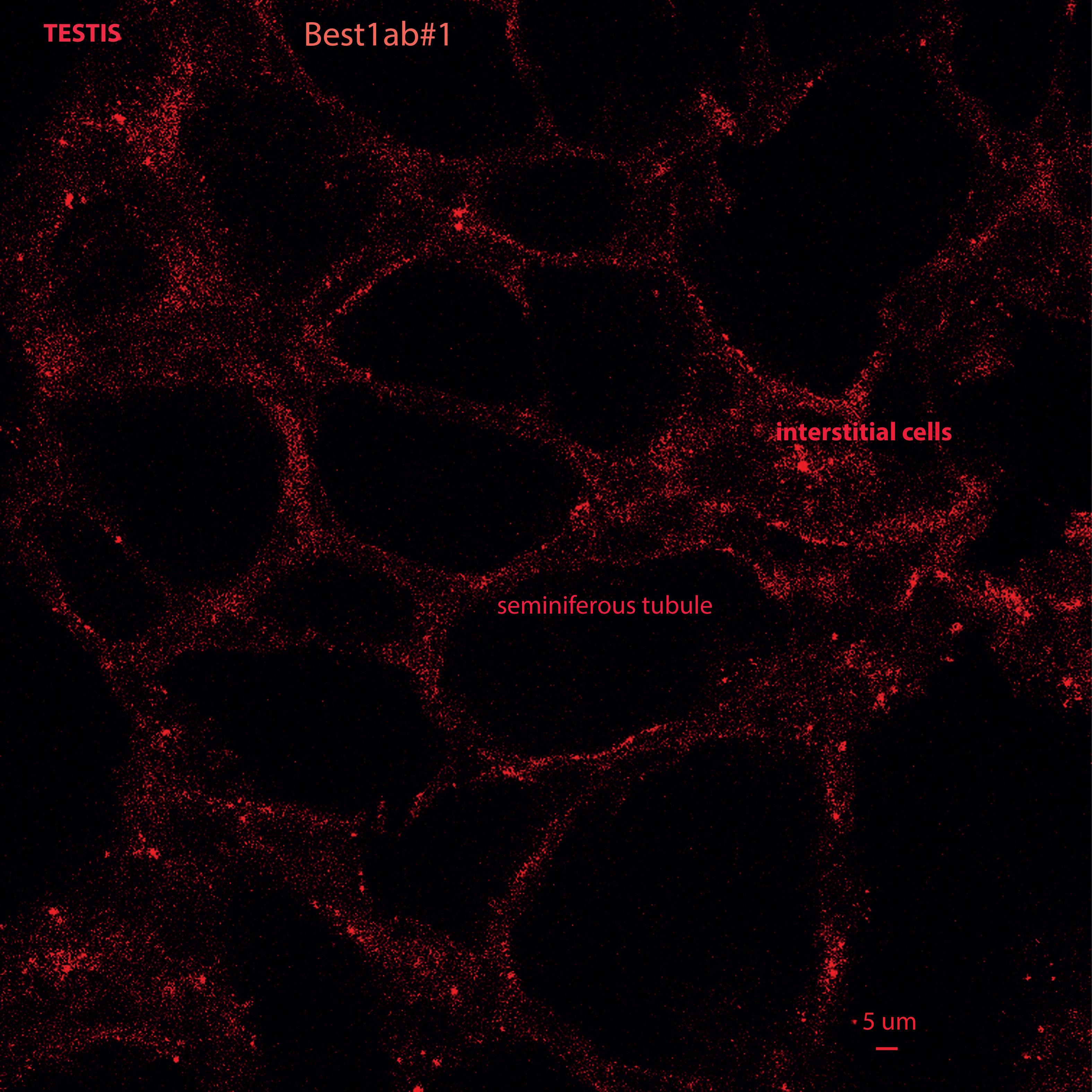

Supplement: Supplementary file 8 [file Image_7.jpeg]

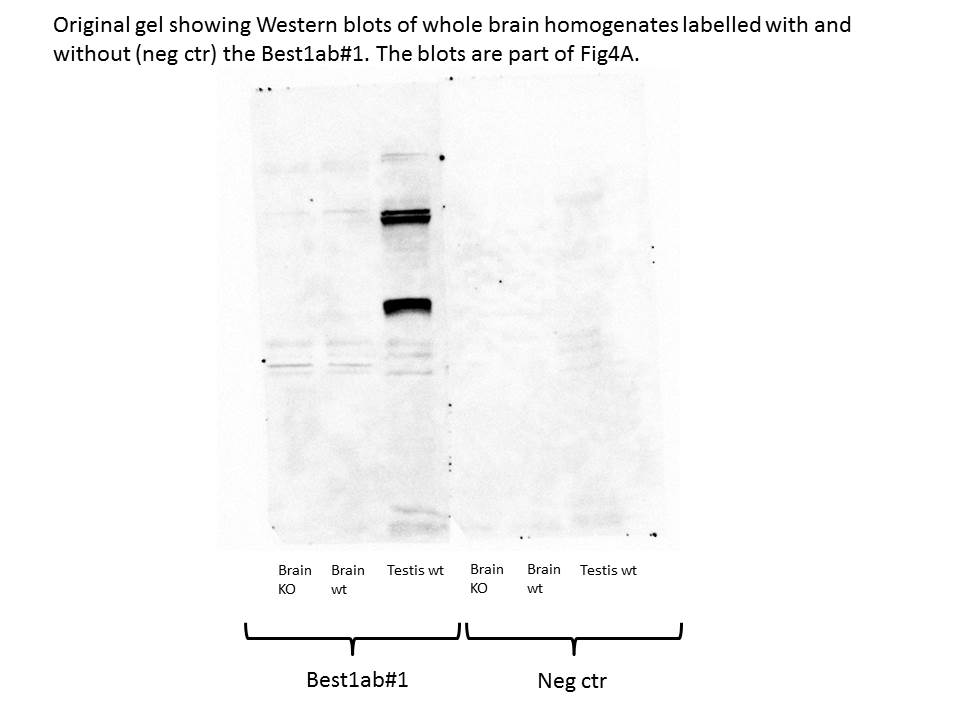

Supplement: Supplementary file 9 [file Image_8.jpeg]

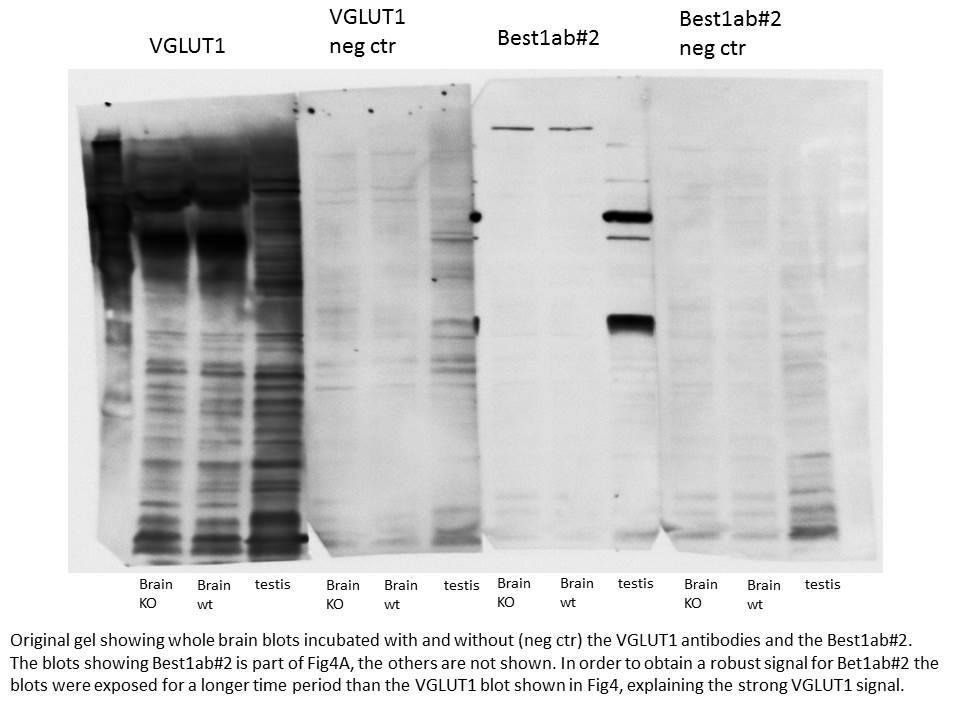

Supplement: Supplementary file 10 [file Image_9.jpeg]

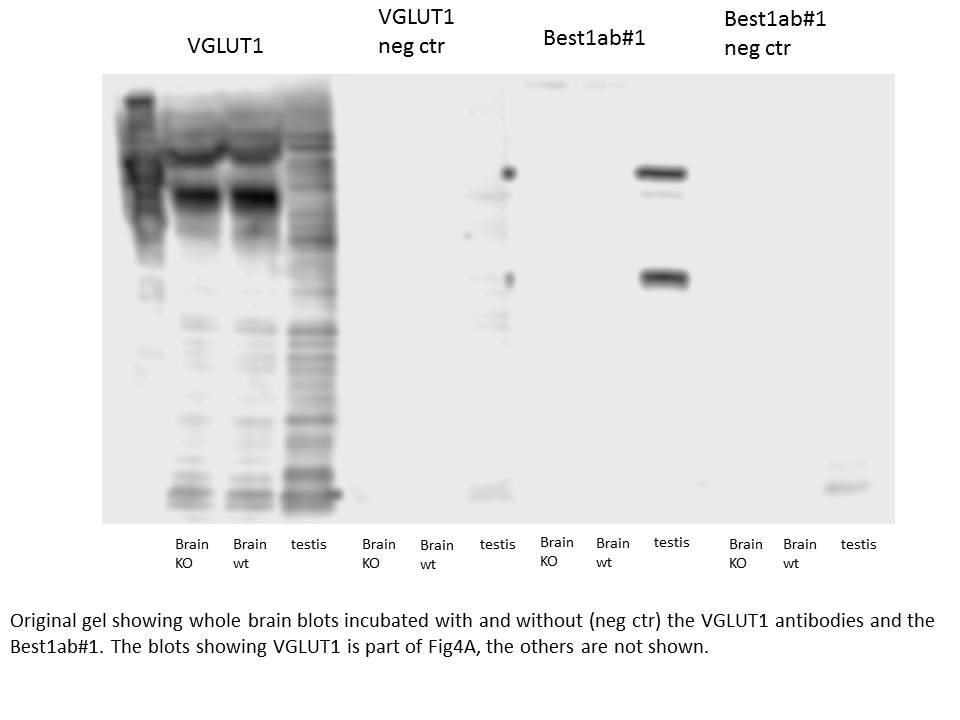

Supplement: Supplementary file 11 [file Image_10.jpeg]

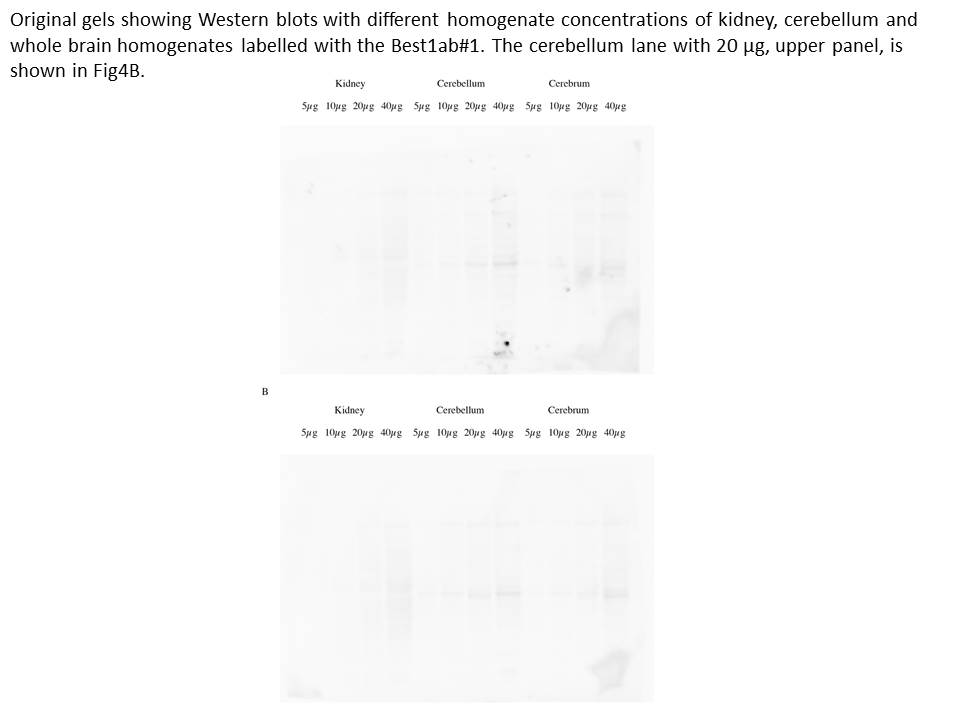

Supplement: Supplementary file 12 [file Image_11.jpeg]
